# Recent Developments of Nanodiamond Quantum Sensors for Biological Applications

**DOI:** 10.1002/advs.202200059

**Published:** 2022-03-27

**Authors:** Yingke Wu, Tanja Weil

**Affiliations:** ^1^ Max Planck Institute for Polymer Research Ackermannweg 10 Mainz 55128 Germany

**Keywords:** cell biology, intracellular nanoscale sensing, nanodiamonds, nitrogen‐vacancy (NV) centers, ultrasensitive diagnosis

## Abstract

Measuring certain quantities at the nanoscale is often limited to strict conditions such as low temperature or vacuum. However, the recently developed nanodiamond (ND) quantum sensing technology shows great promise for ultrasensitive diagnosis and probing subcellular parameters at ambient conditions. Atom defects (i.e., N, Si) within the ND lattice provide stable emissions and sometimes spin‐dependent photoluminescence. These unique properties endow ND quantum sensors with the capacity to detect local temperature, magnetic fields, electric fields, or strain. In this review, some of the recent, most exciting developments in the preparation and application of ND sensors to solve current challenges in biology and medicine including ultrasensitive detection of virions and local sensing of pH, radical species, magnetic fields, temperature, and rotational movements, are discussed.

## Graphical abstract

The recently developed nanodiamond (ND) quantum sensing technology shows great promise for ultrasensitive diagnosis and probing subcellular parameters within living cells at ambient conditions. In this review, some of the recent, most exciting developments of this technology, its potential, and challenges for solving current issues in biology and medicine are critically discussed.

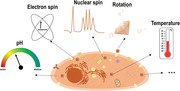

## 1 Introduction

Living biological systems are challenging to understand due to their high structural complexity and dynamics. They are characterized by the existence of, i.e., nanoenvironments, molecular gradients, the formation of transient structures and highly dynamic interactions. Many different species such as biomolecules, ions, and radicals, coexist within the highly crowded and dynamic nanoenvironments within cells. Various biological reactions are constantly proceeding, causing an inhomogeneous distribution of different species, temperatures, and forces at the nanoscale. Local and quantitative detection of these environmental parameters and molecules including transient reactive structures with limited lifetimes will advance our understanding of the living systems. Today, many techniques, such as electron microscopy, scanning tunneling microscopy, and atomic force microscopy, have been developed for the detection of molecules with atomic resolution. However, they are usually limited by strict conditions such as low temperatures, high vacuum, and large sample volumes or they are too invasive or limited to surface characterization, which makes them not suitable for the “warm, wet and noisy” conditions within living cells.^[^ ^1^, ^2^ ^]^

Nanodiamonds (NDs) are considered to be chemically inert because they mainly consist of sp^3^‐hybridized carbon atoms within their lattice and they have been shown to be biologically compatible in several independent studies.^[^ ^3^, ^4^, ^5^ ^]^ Moreover, by introducing other defect atoms (e.g., N, Si, etc.) and vacancy centers into the ND lattice, color centers (i.e., nitrogen‐vacancy (NV) center, silicon‐vacancy (SiV) center) are formed that provide several unique properties such as emission and magneto‐optical features.^[^ ^6^ ^][^ ^7^ ^][^ ^8^ ^]^ The NV center is a point defect consisting of a substitutional nitrogen and a nearby vacancy revealing stable fluorescence without photobleaching or photoblinking, far‐red emission, long lifetime, and high quantum efficiency.^[^ ^9^, ^10^ ^]^ Consequently, NDs with these defects have attracted much attention for bioimaging applications.^[^ ^11^, ^12^ ^]^ The NV center also shows spin‐dependent photoluminescence allowing quantitative detection of electrons and in certain cases even nuclear spins at ambient conditions by optical readout. These unique features open the way to the detection of radicals and chemical reactions at the nanoscale and at ambient conditions, which is particularly attractive for biological applications.^[^ ^13^, ^14^, ^15^ ^]^ Herein, we introduce the physical properties of color centers in NDs, focusing first on the widely used NV center. We then summarize the main preparation techniques of fluorescent NDs and recent improvements. Later, the surface functionalization of NDs is described, which is essential for colloidal stability in aqueous media as well as for controlled nanoparticle membrane interactions to study NDs within living cells. Moreover, the most recent developments, great potential, and challenges of ND sensors in biological systems will be discussed in detail, including ultrasensitive (sub‐picomolar)^[^ ^16^ ^]^ detection of virions and sensing of local parameters such as pH, radicals, magnetic noise, temperature, and 3D rotations. We envision groundbreaking discoveries in the field of ND sensing within biological systems, which will enhance our understanding of the complex and highly dynamic processes and structures within cells.

## 2 Photophysical Properties of Nanodiamonds

**Scheme 1:**
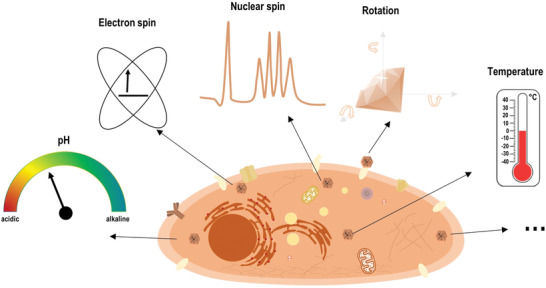
Nanodiamond quantum sensors can detect local parameters such as pH, temperature, nuclear and electron spins within biological systems.

A pure and structurally perfect diamond is colorless and transparent, with a transmittance range from 225 to 2000 nm.^[^ ^17^ ^]^ Occasionally, natural diamonds exhibit different colors due to the presence of chemical impurities and/or structural defects in the diamond lattice. These impurities and/or defects are often termed as color centers that have received much interest due to their exciting photophysical properties. Around 500 color centers have been found in diamond so far.^[^ ^18^ ^]^ Some of these color centers, such as the nitrogen‐vacancy (NV), the silicon‐vacancy (SiV) and the Stuttgart‐1 (ST1) center, could be used for nanoscale sensing due to their spin‐dependent photophysical properties. Their potential for diverse applications and the respective limitations are summarized and discussed in more detail later in this section (**Scheme** **1**).

**Figure 1:**
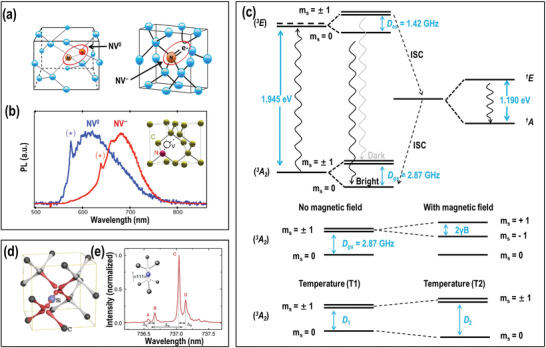
a) Schematic presentation of NV^0^ and NV⁻ centers in a diamond unit cell. Reproduced under the terms of the Creative Commons CC‐BY license.^[^ ^34^ ^]^ Copyright 2017, The Authors. Published be MDPI. b) Normalized photoluminescence spectra of a single NV° center (blue) and NV⁻ center (red) in nanodiamonds (⋆ indicates the zero‐phonon line). Reproduced with permission.^[^ ^35^ ^]^ Copyright 2010, American Physical Society. c) Simplified energy‐level diagram of the NV⁻ center and the influence of the magnetic field and temperature on the splitting and shift of energy levels. d) Schematic of the SiV center. Reproduced with permission.^[^ ^36^ ^]^ Copyright 2014, American Physical Society. e) Fluorescence spectrum at 4 K for a SiV⁻ color center in diamond. Reproduced with permission.^[^ ^37^ ^]^ Copyright 2014, Nature Publishing Group.

The NV center^[^ ^6^ ^]^ is an atom defect comprising a substitutional Nitrogen atom adjacent to a vacancy center in the diamond lattice (see **Figure** **1**a). The Nitrogen atom has five valence electrons, three of which form individual covalent bonds with the surrounding carbon atoms and two of which remain as a lone pair. The vacancy has three unpaired electrons, two of which make a quasi‐covalent bond and one of which remains unpaired. This NV center contains five electrons and is denoted charge neutral NV (NV^0^), with the spin quantum number of 1/2. It can receive an additional electron from the crystal lattice thus forming the negatively charged NV (NV⁻) by changing the Fermi level position. The extra electron forms a spin *S* = 1 pair with the one electron in the vacancy. Both the NV^0^ and NV⁻ are optically active revealing very stable emission that is independent of the size of the ND and no spectral shift occurs, when the size of the NDs increases. They do not bleach under irradiation for months, which is particularly useful for bioimaging.^[^ ^19^ ^]^ In addition, NV⁻ possesses spin‐dependent fluorescence, which allows measuring the various quantities (e.g., temperature, magnetic fields, and electric fields) by optical read out and with unprecedented sensitivity and precision using the concept of optically detected magnetic resonance (ODMR).^[^ ^20^ ^]^ As shown in Figure 1d, the SiV is characterized by a three electronic level structure, comprising the ground state ^3^A_2_, an excited state ^3^E, and a metastable singlet state containing two levels with the symmetries ^1^A and ^1^E.^[^ ^21^ ^]^ The transition from the ground state ^3^A_2_ to the excited state ^3^E has a resonance wavelength of 637 nm (638 nm, zero phonon line, 1.945 eV) and it can be efficiently excited by most lasers with wavelengths below 640 nm. Major emission appears in the sidebands between 630 and 800 nm due to vibration (Figure 1b), and only a small number of photons are emitted into the zero phonon line. Importantly, the ground and excited states are spin‐triplet transitions, and they further split into three spin sublevels, *m*_s_ = 0 and *m*_s_ ± 1, where the two *m*_s_ = ±1 states are degenerated and have higher energy than the *m*_s_ = 0 state (Figure 1c). The zero‐field splitting is 2.87 GHz in the ground state and 1.42 GHz in the excited state.^[^ ^6^ ^]^ Under resonant optical excitation, the electrons in the ground state are raised to the excited state and then return to the ground state via two possible pathways (1) by the emission of a photon of 637 nm (638 nm) in the zero phonon line (ZPL) and phonon sideband or (2) via the aforementioned metastable singlet states through intersystem crossing (ISC) with the weak infrared emission of a 1042 nm (1.190 eV) photon. The latter pathway preferentially returns to the *m*_s_ = 0 state resulting in a decrease in the emitted fluorescence intensity of around 30%. Pumping with a resonant microwave frequency at 2.87 GHz promotes the electrons to the degenerated *m*_s_ = ± 1 states. By applying a magnetic field, the degenerated *m*_s_ = ± 1 is lifted, causes Zeeman splitting and the fluorescence decreases at two resonant frequencies, given by 2*γB*, where *γ* = 2*π* × 28 GHz/T is the electron gyromagnetic ratio and B is the magnetic field parallel to the NV axis. Moreover, the zero‐field splitting of the NV⁻ center is temperature‐dependent and the resonant frequencies shift by −74 kHz K^−1^.^[^ ^22^ ^]^ Furthermore, the relaxation time (*T*_1_) of the NV⁻ is also sensitive to the external noise, which can be used for sensing. A detailed photophysical discussion of the unique properties of the NV center and the related sensing applications has been included in reviews by Schirhagl et al. in 2014,^[^ ^23^ ^]^ by Wu et al. in 2016,^[^ ^24^ ^]^ and by Mzyk et al.,^[^ ^25^ ^]^ and Zhang et al. in 2021.^[^ ^26^ ^]^

In addition to the more established NV‐based quantum sensor, there are also other color centers with similar photophysical properties suitable for ODMR and quantum sensing. For instance, the SiV center is formed by replacing two neighboring carbon atoms in the diamond lattice with one silicon atom situated between the two vacant lattice sites. It has a very sharp zero‐phonon line (ZPL) at 738 nm (1.68 eV) and a minimal phonon sideband at around 766 nm. Approximately 70% of its emission returns to the ZPL, compared to only 4% of the NV center (Figure 1e). The temperature‐dependent shift of the sharp ZPL of SiV in ND is much more sensitive than that of the NV in ND and enables an all‐optical temperature measurement, without the application of microwave radiation.^[^ ^27^, ^28^ ^]^ Recently, the ODMR of SiV centers has been realized for negatively charged SiV (SiV⁻)^[^ ^8^ ^]^ and neutral SiV (SiV^0^) centers,^[^ ^29^ ^]^ although the fine structure of the excited state is poorly understood and off‐resonant spin polarization is limited. The SiV center has been discussed as ND quantum sensor providing many attractive features for biological applications such as very stable emission in the near‐infrared region of light beyond the autofluorescence of cells and the capacity to detect temperature and other parameters by direct optical readout. The number of SiV in NDs is not as high as the occurrence of NV in NDs. The NDs with the size of 60 nm can contain more than 100 NV. According to the recent study,^[^ ^30^ ^]^ the recently synthesized 60 nm NDs only has 4–5 SiVs and shows around 600 kcps. Besides, they have been used for live‐cell dual‐color imaging and intracellular thermometry. Currently, no standard procedure exists for the preparation of NDs with similar sizes and numbers of SiV comparable to NV containing NDs.

The recently identified new defect center in diamond, known as the Stuttgart‐1 (ST1) center,^[^ ^31^, ^32^, ^33^ ^]^ provides a singlet electronic ground state and a photoexcited triplet state. It also shows spin‐dependent fluorescence, and the spin readout contrast of the ST1 centers is even higher than that of the NV center, making it a promising candidate for the next generation of quantum sensors. However, its structure and chemical composition are still unknown and have been detected only three times.^[^ ^31^, ^32^, ^33^ ^]^

## 3 Preparation and Functionalization of Nanodiamond Quantum Sensors

### 3.1 Preparation of Nanodiamond Quantum Sensors

Over the past decades, many different strategies have been developed for the preparation of NDs, including the detonation technique,^[^ ^38^ ^]^ high‐pressure high‐temperature (HPHT) synthesis^[^ ^39^ ^]^ and the related seed‐growth HPHT preparation method,^[^ ^40^ ^]^ laser ablation,^[^ ^41^ ^]^ ultrasound cavitation,^[^ ^42^ ^]^ chlorination of carbides,^[^ ^43^ ^]^ cold‐compression synthesis,^[^ ^44^ ^]^ and microfabrication.^[^ ^45^ ^]^ Among them, only the first three methods can be used to obtain larger‐scale NDs batches, i.e., for commercialization.^[^ ^46^ ^]^ In addition, ideal NDs for quantum sensing applications should have a controlled number of very stable color centers at spatially defined locations within their lattice.

**Figure 2:**
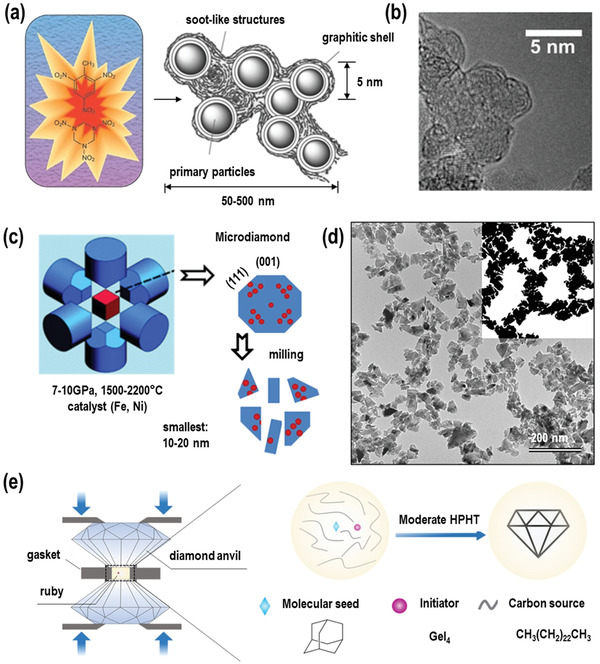
a) Detonation NDs produced by detonation of carbon‐containing explosives; diamond‐containing soot is collected from the bottom and the walls of the chamber. Left: Reproduced with permission.^[^ ^62^ ^]^ Copyright 2012, Nature Publishing Group. Right: Reproduced with permission.^[^ ^63^ ^]^ Copyright 2007, Wiley‐VCH. b) High‐resolution TEM image of pristine detonation NDs. Reproduced with permission.^[^ ^63^ ^]^ Copyright 2007, Wiley‐VCH. c) NDs produced by the HPHT methods. Reproduced with permission.^[^ ^55^ ^]^ Copyright 2015, American Vacuum Society. d) TEM image of HPHT NDs. Reproduced with permission.^[^ ^64^ ^]^ Copyright 2014, Elsevier. e) Illustration of the GeI_4_‐mediated synthesis of nanodiamonds under mild HPHT conditions. Reproduced with permission.^[^ ^40^ ^]^ Copyright 2020, Elsevier.

Until now, NDs prepared by laser ablation containing color centers have not been reported and therefore, they cannot be used for bioimaging or sensing applications. Detonation nanodiamonds (DNDs) are also considered less suitable for quantum sensing applications because they are usually polycrystalline and ultra‐nanocrystalline with only a few nanometers of crystalline regions, which were considered less suitable to host stable color centers such as NV⁻ center due to the large surface tension.^[^ ^47^ ^]^ DNDs are prepared upon detonation of a mixture of explosives (**Figure** **2**a), e.g., trinitrotoluene (TNT) and hexogen (RDX), in a sealed metal chamber where supersaturated carbon vapor from the explosives condenses into liquid droplets and then crystallizes into 3–5 nm NDs as temperature and pressure decrease. The products collected from the wall and the bottom of the chamber are diamond soot containing approximately 75 wt% NDs and 25 wt% other carbon allotropes or impurities, resulting in stable aggregates with sizes of 50–500 nm. DNDs naturally contain high concentrations of NV centers. However, without the removal of the graphitic material around the diamond core, NV fluorescence is generally not observed. Although NV fluorescence has been demonstrated in a few isolated DNDs, their emission is generally weak and often shows blinking.^[^ ^48^ ^]^ In 2017, a few bright and photostable NV⁻ centers and an ODMR spectrum from unprocessed aggregates of DNDs were reported by Gibson and co‐workers^[^ ^49^ ^]^ and the ODMR spectra of single bright NV⁻ centers in aggregates of DNDs were reported by I. I. Vlasov, et al.^[^ ^50^ ^]^ The aggregates of DNDs with NV⁻ centers were used as nanothermometer by Shirakawa and co‐workers^[^ ^51^ ^]^ and later, monodispersed DNDs with about 5 nm dimensions (Figure 2b) and relatively stable NV⁻ centers have been reported by the same group.^[^ ^52^ ^]^ Monodispersed DNDs offer many attractive features allowing their application as quantum sensors, and they could emerge as particle‐of‐choice in bioimaging and sensing in case their synthesis would be scalable and more cost‐effective than the nowadays available methods. In addition, DNDs have more regular shapes and they are much smaller than HPHT NDs. The small size of detonation NDs could in principle allow the preparation of NDs with only a single stable NV center, which could pave the way to the production of NDs with single NV and more uniform shapes. This may provide us with more uniform properties, such as narrow *T*_1_ time distribution, compared to current HPHT NDs.

Most NDs currently used for quantum sensing are produced by high‐energy ball milling of HPHT microdiamonds. The HPHT microdiamond synthesis proceeds under high pressure (7–11 GPa) and high temperature conditions (1500–2200 ⁰C) in a hydraulic press in the presence of metal catalysts (Fe, Ni, or/and Co) (Figure 2c).^[^ ^53^, ^54^ ^]^ In the HPHT reactor, N_2_ gas is first dissolved in the solvent metal and then introduced into the diamond lattice during subsequent crystal growth. In the resulting microdiamond lattice, the N atoms remain isolated and are bound to four other C atoms. Two strategies have been reported to generate NDs with NV^–^centers. In the first strategy, the HPHT microdiamonds are irradiated to create vacancies in the lattices. They are then annealed at elevated temperature (e.g., 900 °C) to form the NV centers, mechanically milled to obtain NDs and then treated in a highly oxidative environment to remove surface impurities.^[^ ^55^ ^]^ Nowadays, this method is widely used to produce the commercially available NV NDs. However, there are also a few limitations associated with this approach as some of the NV centers are destroyed during the milling process, which makes it very challenging to prepare NDs with high NV content. Therefore, an extremely high number of NV centers in the starting microdiamond precursor material is required, which still represents a great challenge to prepare. The second strategy is to first mechanically mill the HPHT microdiamonds to NDs, and then irradiate, anneal, and decontaminate the NDs with an oxidative treatment. The thus produced NVs are typically stable, but it is difficult to create NV color centers in all NDs.

In the above mentioned ND preparation methods, the amount and location of the NV centers in NDs cannot be precisely controlled, and each ND has different and irregular shapes.^[^ ^56^ ^]^(see Figure 2d) For quantum sensing, NDs should ideally contain a distinct number of shallow NVs closer to the ND surface and their shapes and surface functionalities should be as uniformly as possible to significantly improve the sensing accuracy.^[^ ^54^ ^]^ In particular, the preparation of a ND with a single NV situated in the center of the ND would be very attractive as single spin probe. To date, one of the most promising approaches to produce such NDs is based on the HPHT method (see Figure 2e). Here, small organic molecules such as diamondoids could be used as precursors (or “seeds”) instead of small crystals and exposed to HPHT reaction conditions. This so‐called “seeded growth” based on organic precursor molecules could potentially enhance nucleation to form higher‐quality NDs with distinct defects. Pre‐organization of the defect atoms in the organic precursor molecules could potentially provide new opportunities for the controlled growth of distinct color centers within the ND lattice. For example, the synthesis based on adamantyl‐precursor molecules containing Nitrogen or Silicon atoms could provide access to NDs with defined numbers of NV or SiV centers. However, to ultimately achieve molecular preorganization of the atom defects, the temperature during the HPHT reaction has to be as low as possible to avoid decomposition of the organic precursor molecules. Adamantane derivatives have attracted great interest as precursors for the synthesis of micro or nano‐diamonds, as its diamond‐like structure could serve as “building block” or so‐called molecular “seed” for the formation of the diamond lattice. As reported by R. H. Wentorf Jr. almost 60 years ago,^[^ ^57^ ^]^ diamond could be synthesized from adamantine at 15 GPa and 2000 °C under the same conditions as from other hydrocarbons. However, in this study, ND synthesis was most likely accomplished by the recrystallization of a pyrolysis intermediate of graphite rather than the direct conversion of adamantane to diamond by the nucleation and growth procedure. Successful synthesis of aggregated NDs below the direct graphite‐diamond transition condition was first reported by Morigami and co‐workers in 1992.^[^ ^58^ ^]^ In 2014, the direct production of doped ultrasmall diamonds from 9‐borabicyclo[3,3,1]nonane dimer applying HPHT was published by I. I. Vlasov, et al.^[^ ^59^ ^]^ At the same time, the HPHT synthesis of NDs with NV centers and SiV centers based on mixtures of hydrocarbon, fluorocarbon, and organic silicon compounds was reported by Agafonov and co‐workers^[^ ^60^ ^]^ In 2017, the first successful implementation of mass synthesis of isolated NDs from adamantane below the conditions for direct conversion of graphite into diamonds was reported by I. I. Vlasov, et al.^[^ ^61^ ^]^ To further decrease the pressure and temperature during HPHT synthesis, GeI_4_ was applied as catalysis for ND synthesis at only 3.5 GPa and 500 °C, which is much lower than the commonly reported HPHT NDs synthesis conditions.^[^ ^61^ ^]^ However, the underlying mechanisms of the HPHT synthesis of NDs have to be elucidated to ultimately achieve NDs of fully controllable sizes with distinct numbers of color centers at defined locations.

### 3.2 Functionalization of Nanodiamond Quantum Sensors for Biological Applications

**Figure 3:**
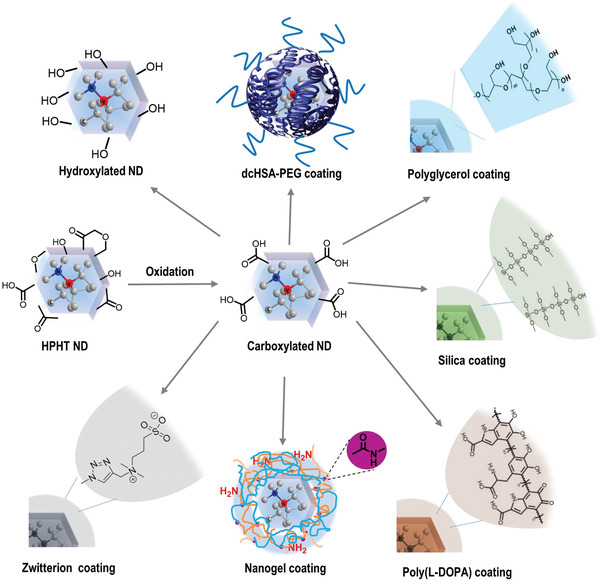
Schematic presentation of surface functionalization of NDs. The prepared HTHP NDs provide various functional surface groups, and acid oxidative treatment (HClO_4_: HNO_3_: H_2_SO_4_ = 1: 1: 1) produces NDs with carboxylic groups. The carboxylic acid surface groups can be subsequently reduced to hydroxyl groups^[^ ^68^ ^]^ or modified with zwitterions^[^ ^100^ ^]^ or coated by a protein‐based polymer (such as the modified serum albumin‐PEG copolymer dcHSA‐PEG),^[^ ^72^ ^]^ polyglycerol,^[^ ^81^ ^]^ silica,^[^ ^88^ ^]^ poly(l‐DOPA),^[^ ^95^ ^]^ and polymer nanogel shell.^[^ ^98^ ^]^ ND scheme reproduced with permission.^[^ ^98^ ^]^ Copyright 2021, Wiley‐VCH. dcHSA‐PEG scheme reproduced with permission.^[^ ^72^ ^]^ Copyright 2015, Wiley‐VCH.

Surface functionalization of ND quantum sensors is a crucial step to impart stability and to engineer the nano‐bio‐interface for imaging and quantum sensing applications in cellular environments. Even though detonation NDs provide more regular shapes and they are much smaller than HPHT NDs, they have not been used as quantum sensors for biological applications, most likely due to the fact that they are not commercially available and no standardized and reproducible production method is currently available. Therefore, the following part only reviews surface functionalization strategies of NDs prepared by HPHT method. However, in principle, these surface functionalization strategies can be used for the NDs produced by other methods as well. Although, all nanodiamonds irrespective of their production method are purified and treated via the different procedures, oxidative treatment is the main and most widely used procedure to purify the NDs, and is the current used procedure to produce the commercially available carboxylated NDs aqueous solution. In the case of detonation NDs, water or ice is applied for the cooling, which results in the reaction with highly reactive hydroxyl species. Moreover, the further purification of NDs is most often using oxidizing acids and/or air oxidation to remove the metal impurities, soots and sp^2^ carbons.^[^ ^65^, ^66^, ^67^ ^]^ The oxidative treatment leads to the formation of carbonyl and carboxyl groups on the surface of NDs. In the HPHT method, after mechanically milling of the microdiamonds to obtain NDs, the oxidative treatment is adapted to remove surface impurities and get carboxylated NDs. More detailed discussion about the purification procedure can be found in the review.^[^ ^68^ ^]^ In brief, ND emitters with stable color centers and negatively charged carboxylic acid groups are usually obtained that can be dispersed in water and used for further modification.^[^ ^55^ ^]^ Surface functionalization is important to further enhance the colloidal stability of NDs in biological buffers or cell media and to prevent their aggregation or precipitation. In addition, cell targeting molecules could be introduced to enable cellular uptake.^[^ ^69^ ^]^ However, functionalization should not increase the hydrodynamic radius of NDs too much as this would decrease their sensitivity.^[^ ^70^ ^]^ Various ND surface coating materials and strategies have been reported and some of the most prominent ones are summarized in **Figure** **3**.

NDs are non‐covalently modified with macromolecules by taking advantage of their large relative surface area and electrostatic interactions. Several (bio)polymers have been applied as ND surface coatings due to many non‐covalent interactions between the polymer and the ND surface. In this way, the proteins insulin,^[^ ^71^ ^]^ serum albumin,^[^ ^11^ ^]^ and albumin‐based copolymers,^[^ ^72^ ^]^ virus proteins^[^ ^73^ ^]^ or the synthetic polymers polyethyleneimine,^[^ ^74^ ^]^ and poly‐l‐lysine^[^ ^75^ ^]^ have been adsorbed on the ND surface. The non‐covalent functionalization is often more straightforward and is based on van der Waals interactions as well as electrostatic interactions of the biomolecules and the nanodiamond surface. Therefore, this approach proceeds well if biomolecules with net positive charged surface are adsorbed onto the negatively charged NDs, whereas polyanionic biomolecules such as DNA are mostly attached via covalent conjugation strategies. The stability of the newly formed shell consisting of biomolecules still needs to be studied on a case by case basis. The long‐term stability of NDs with adsorbed surface molecules could be limited under physiological conditions due to ligand exchange or ligand loss as the interaction between the ND surface groups and biomolecules is affected by various parameters such as temperature, pH, and ionic strength.

Covalent modification offer certain advantages to permanently conjugate the desired molecules, and several different strategies have been reviewed previously.^[^ ^68^, ^76^ ^]^ Initially the NDs were covalently modified with water‐soluble small molecules and the carboxylic acid surface groups of the NDs were functionalized to improve the colloidal stability in biological buffer. However, aggregation and precipitation of the NDs were often observed during these reactions.^[^ ^77^, ^78^ ^]^ Water‐soluble polymers such as polyethyleneglycol (PEG) were also used to modify NDs and colloidal stability was significantly improved in water.^[^ ^78^, ^79^ ^]^ However, due to the low PEG grafting density, PEG‐modified NDs are not sufficiently stable in biological buffers due to the limited number of reactive carboxylic acid surface groups.^[^ ^79^, ^80^ ^]^ Branched polyglycerol‐modified NDs were developed by Komatsu and co‐workers^[^ ^81^ ^]^ Here, the glycidol precursors reacted with the hydroxyl and carboxylic acid surface groups in a “grafting from” ring‐opening polymerization to form the branched polyglycerol shell at the nanodiamond surface. These polyglycerol modified NDs show excellent colloidal stability in various biological buffers and many hydroxyl groups on the surface allow further post‐functionalization. Due to their high solubility, they can be purified by liquid chromatography and characterized by NMR spectroscopy. However, even though low yields in the production of single ND have somewhat limited the application of polyglycerol ND coatings, several exciting biological applications have been reported.^[^ ^82^, ^83^, ^84^, ^85^ ^]^ The loss of structure integrity and function of biomolecules could occur via both covalent and non‐covalent surface functionalization strategies. Therefore, both strategies are still being used in the community without one being superior to the other. At present, the attachment of the desired biomolecule needs to be tested and often, the conventional bioconjugation between carboxylic acid groups at the ND surface and primary amine groups of the biomolecules are linked applying standard coupling reagents. Moreover, the site‐selective modification strategy of biomolecules (i.e., peptides, antibody) represents an emerging strategy to display bioactive epitope on the nanoparticle surface without affecting their bioactivities. A recent review summarised how site‐selective dual functionalization of proteins can be performed without affecting their functions.^[^ ^86^ ^]^

Silica (SiO_2_) coating has also been used to modify the surface of NDs,^[^ ^9^ ^] [^ ^87^, ^88^ ^]^ which also allows post‐modification to further functionalize the silica‐coated NDs. The silanol groups on the surface of these coatings can enhance the colloidal stability of the resulting ND suspensions over a broader range of pH values or electrolyte concentrations.^[^ ^9^ ^]^ Moreover, the silica coating is optically transparent^[^ ^89^ ^]^ and allows efficient transmission of excitation and emission light. In addition, the irregular shape of the NDs changes to egg‐like spheroids after silica coating. However, this coating is not stable over longer time periods and after purification, free silanes are removed from the silica‐coated ND dispersion, and the polycondensation reaction proceeds reversely, resulting in the hydrolysis of the silica shell.^[^ ^90^, ^91^ ^]^ Notably, weakly crosslinked functionalized silanes are even more prone to hydrolyze the original silica shell.

Inspired by mussel foot proteins, the neurotransmitter dopamine forms a highly adhesive biopolymer^[^ ^92^ ^]^ which has been applied as surface coating of many different nanoparticle surfaces including NDs.^[^ ^93^, ^94^, ^95^ ^]^ The polydopamine coating provides many functional groups including amines, alcohols, and conjugated Michael acceptors for post‐functionalization. However, polydopamine coatings could not prevent ND aggregation, especially in physiological buffer conditions^[^ ^96^ ^]^ and post‐functionalization with, e.g., poly(ethylene glycol)^[^ ^97^ ^]^ or proteins^[^ ^95^ ^]^ as stabilizers was crucial. Moreover, polydopamine strongly absorbs the excitation light, and the fluorescence of the NV in NDs decreases dramatically with increasing thickness of the polymer shell. In consequence, the signal readout was strongly decreased even for only a few nanometers of shell thickness, which was a major limitation for sensing applications.^[^ ^95^ ^]^

We have recently reported an adsorption and crosslinking procedure to generate a nanogel shell around NDs.^[^ ^98^, ^99^ ^]^ First, the adsorption of multifunctional and positively charged ligands precoated the NDs based on electrostatic interactions, followed by a cross‐linking step to afford a stable and soft crosslinked nanogel shell. In this work, highly branched (hyperbranched) polyethyleneimine (PEI), a cationic polymer with multiple primary amino groups, was adsorbed to the ND surface, before the 4‐armed polyethyleneglycol cross‐linker reacted with PEI to form the nanogel. The nanogel (NG) shell thickness was as low as 10 nm in aqueous solution, which enabled quantum sensing applications and the ND‐NGs display a long‐term stability. ND‐NG were further modified with paramagnetic ions such as Gd^3+^, the iron‐containing protein cage ferritin, the photothermal reagent indocyanine green, as well as multiple Ruthenium complexes the photodynamic agent.

## 4 Biological Applications of ND Quantum Sensors

NDs are physically and chemically stable under ambient conditions due to their sp^3^‐hybridized carbon lattice. Moreover, they can be considered biocompatible according to cell and animal experiments,^[^ ^3^, ^4^, ^5^ ^]^ which is a prerequisite for their application as sensors in biology. Due to their stable emission, large surface‐to‐volume ratio and excellent biocompatibility, NDs were first used as high performance emitters for bioimaging and stimulated emission depletion (STED) microscopy in the living cell,^[^ ^101^ ^]^ and as a platform for drug delivery in cancer therapy.^[^ ^69^ ^]^ In the past years, the sensing properties of NDs have received much attention for acquiring local information in biological systems. Currently, all of the quantum sensing applications in biology are carried out by HPHT NDs containing NVs, mainly because of the relatively mature manufacture process and the fact that they are commercially available.

### 4.1 Spin‐Enhanced Nanodiamond Biosensing for Ultrasensitive Diagnosis

**Figure 4:**
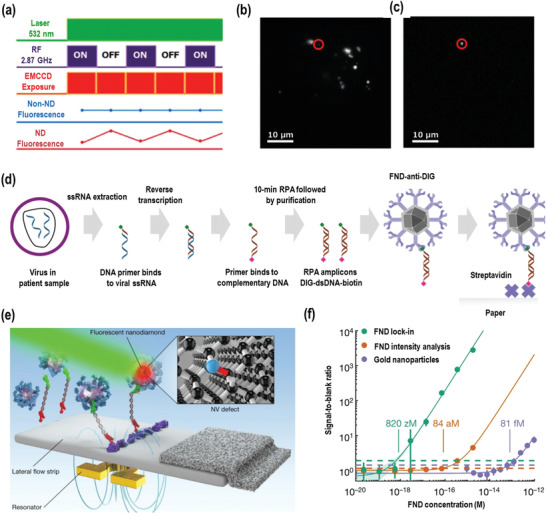
a) Time chart of fluorescence excitation at 532 nm microwave irradiation, and image acquisition used in the selective imaging protocol, along with the expected profiles of non‐ND and ND fluorescence. b) A conventional fluorescence image of NV^–^ ND. c) Image after applying the selective imaging protocol. Reproduced with permission.^[^ ^102^ ^]^ Copyright 2012, American Chemical Society. d) A schematic of the assay. Digoxigenin (DIG) and biotin‐modified primers were used in a reverse transcriptase–recombinase polymerase amplification (RT–RPA) reaction to produce labeled amplicons, which bind to anti‐DIG‐functionalized FNDs and streptavidin‐printed test lines on the LFA strips, forming a sandwich structure in the presence of amplicons. e) Illustration of the concept of using NDs in an LFA. The binding of DNA modifications causes NDs to be immobilized at the test line in a sandwich‐format LFA. The inset shows the atomic structure of an NV⁻ center, the origin of FND fluorescence. An omega‐shaped stripline resonator applies a microwave field over the LFA, modulating the fluorescence intensity. f) Comparison of the detection limit. Reproduced with permission.^[^ ^106^ ^]^ Copyright 2020, Springer Nature Limited.

In Chapter 2, it was introduced that the fluorescence of NDs containing NV⁻ centers can be selectively modulated by spin manipulation using microwaves^[^ ^102^, ^103^ ^]^ and magnetic fields,^[^ ^104^, ^105^ ^]^ which enables to separate the signal of NDs in environments with high‐background noise such as auto‐fluorescence of the cells. One of the works from Igarashi et al. is illustrated in **Figure** **4**a,b,c,^[^ ^102^ ^]^ which integrates the gated microwave irradiation and synchronized image acquisition by EMCCD camera. Pulsed microwave irradiation modulates the fluorescence intensity of NDs, whereas non‐ND fluorescence is not affected by microwave irradiation so that the ND signals can be distinguished. There is a great interest in the ultrasensitive detection of virus particles. Recently, a new ultrasensitive quantum diagnostics platform based on the spin manipulation of NDs has been reported by B. S. Miller.^[^ ^106^ ^]^ The sensitivity of the most commonly used tests, paper microfluidic lateral flow assays (LFAs), is usually limited by the background fluorescence of the sample, the substrate, or the readout technique in practical applications. The authors have used the spin‐dependent photoluminescence of NDs in an LFA format for in vitro diagnostics as depicted in Figure 4d,e,f. They achieved a detection limit of 82 × 10^−21^ m (zeptomolar, 10^−18 ^mol L^−1^) in a biotin‐avidin model system, which is 10^5^ times more sensitive than using gold nanoparticles as fluorescence label. Single‐copy detection of human immunodeficiency virus type 1 (HIV‐1) RNA was achieved with the addition of a 10‐min isothermal amplification step. However, the potential concern is the chemical stability of biotin‐avidin conjugates with a dissociation constant (*K*_d_) in the order of ≈10^−14^ mol L^−1^,^[^ ^107^ ^]^ which could possibly dissociate at so low detection concentrations (10^−18 ^mol L^−1^).

### 4.2 Nanoscale pH Sensing with NDs

In living cells, the local pH plays an important role, and its intracellular distribution shows nanoscale spatial inhomogeneities, which affects various biochemical processes and interactions.^[^ ^108^ ^]^ For example, the local pH could contribute substantially to the ATP synthesis and the regulation of intra‐ and intercellular signals.^[^ ^109^, ^110^ ^]^ Therefore, the development of a reliable nanoscopic pH meter that could measure the intracellular pH locally would be crucial to shed light on important cellular processes.

**Figure 5:**
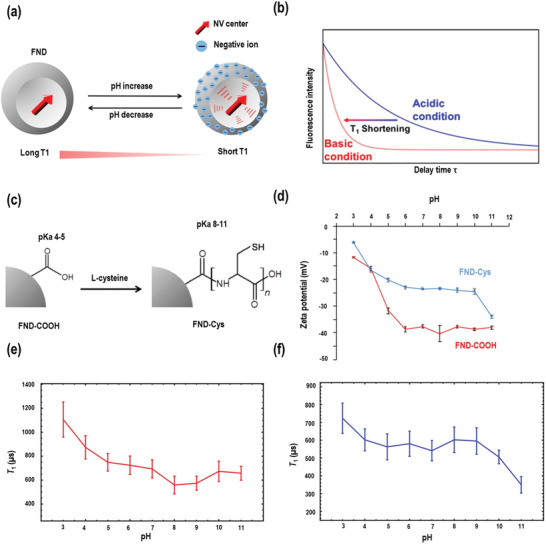
a) *T*_1_‐shortening effect of increasing pH. The surface groups of the ND‐COOH are negatively charged at higher pH. b) Fluorescence intensity plots against the delay time *τ* at different pH. c) Preparation of FND‐Cys. d) pH dependences of FND‐COOH (red) and FND‐Cys (blue) on the zeta potential. e) Typical result of the pH dependence of *T*_1_ of FND‐COOH. f) Typical result of the pH dependence of *T*_1_ of FND‐Cys. Reproduced with permission.^[^ ^112^ ^]^ Copyright 2019, American Chemical Society.

The application of NDs with NV⁻ centers for pH sensing is limited because none of the spin resonance frequency and transversal relaxation time *T*_2_ of NV⁻ centers have shown any response to pH changes. In first experiments by Cigler and co‐workers in 2017,^[^ ^111^ ^]^ NDs were modified with pH‐cleavable linkers functionalized with paramagnetic Gd‐DOTAs complexes as enhancers of spin relaxation. The pH nanosensors prepared by this method responded to pH changes but the pH‐induced linker cleavage was irreversible and could not be used repeatedly. In 2019, Igarashi and co‐workers^[^ ^112^ ^]^ developed a new ND‐type pH meter based on the spin relaxation time *T*_1_ of the NV⁻ centers, which is sensitive to the surface charge of NDs.^[^ ^113^, ^114^ ^]^ (see **Figure** **5**).

The carboxylated NDs are electroneutral in acidic solution and convert into negatively charged carboxylate ions in alkaline solution, resulting in a negatively charged surface.^[^ ^112^ ^]^ (Figure 5a) The negative surface charges perturb the electrical field in the ND and shorten the longitudinal electron‐spin relaxation time *T*_1_ of the NV centers. (Figure 5b) As shown in Figure 5e, the carboxylated NDs showed s significant *T*_1_ shortening with pH increase in acidic conditions but they failed to detect the pH change in alkaline conditions. To solve this challenge, the carboxylic acid groups on the ND surface were modified with poly(l‐cysteines), which have thiol groups with a higher p*K*_a_ than the carboxyl groups. (Figure 5c) The thiol groups are electroneutral at pH 7 and convert to negatively charged thiolate ions at pH 11. (Figure 5d) The poly(l‐cysteines)‐modified ND showed short *T*_1_ in alkaline solution and stable *T*_1_ time in acidic conditions (Figure 5f). However, further improvements are required for measuring the pH within cells, as the intracellular environment is full of different radicals and paramagnetic species, which can also significantly shorten the *T*_1_ time. However, further improvements are required for measuring the pH within cells, as the intracellular environment contains various radicals and paramagnetic species, which can also significantly shorten the T1 time. At present, pH sensing with nanodiamonds is based on the protonation of the surface shell of these nanoparticles. In order to provide specificity and sensitivity, one possible option is to design a soft, thin and semi‐permeable polymer shell that allows diffusion of H^+^ but prevents access of radicals and paramagnetic species that could interfere with the measurement.

### 4.3 Paramagnetic Species Sensing with NDs

**Figure 6:**
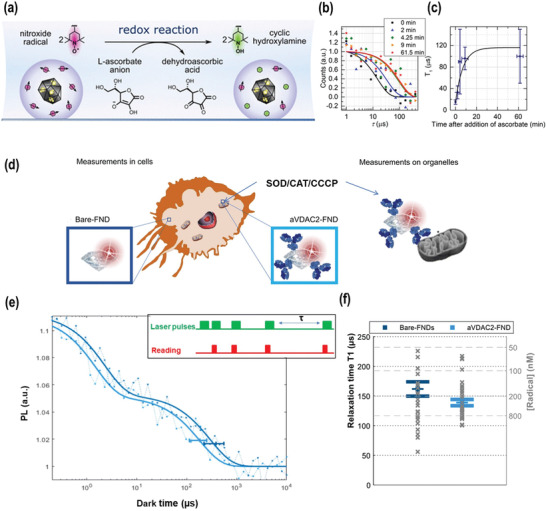
a) Schematic presentation of ND coated with a polymer shell containing spin probes coupled magnetically with NV centers. The radicals in the nanosensor are gradually reduced by a reductant until they are all removed. b) Determination of the *T*_1_ relaxation time of single ND1–ND5 particles. c) The dependence of the average *T*_1_ relaxation time of NV centers on the load of stable nitroxide radicals on the polymer interface of NDs. Reproduced with permission.^[^ ^13^ ^]^ Copyright 2020, American Chemical Society. d) Diamond magnetometry in mitochondria of living cells. First, the free radical formation was investigated in two subcellular locations, uncoated FNDs localized in the cytosol and VDAC2 antibody‐coated NDs that associate with mitochondria. Later the free radicals were measured on single isolated mitochondria. All of the measurements were carried out before and after stimulation with the inhibitor of oxidative phosphorylation carbonyl cyanide 3‐chlorophenylhydrazone (CCCP) at different concentrations as well as the antioxidant enzymes, catalase (CAT, final concentration of 1000 U mL^−1^) and superoxide dismutase (SOD, final concentration of 600 U mL^−1^), both of which can convert radicals to H_2_O_2_ and H_2_O. e) The *T*_1_ relaxation curve is generated from different dark times plotted against the fluorescence intensity. f) Statistical distribution (mean and SE) of *T*_1_ from 26 and 36 NDs. Reproduced with permission.^[^ ^15^ ^]^ Copyright 2021, American Association for the Advancement of Science.

Paramagnetic species such as radicals and metalloproteins play important roles and are widely distributed in the biological system. For instance, DNA‐centered radicals are considered as a major cause for carcinogenesis and aging by triggering genomic damage. Hydroxyl radicals are very reactive oxygen species that can react rapidly with DNA and damaging the DNA irreversibly, which causes cell death.^[^ ^115^, ^116^ ^]^ Ferritin is a universal intracellular protein that stores and releases iron controllably in prokaryotes and eukaryotes.^[^ ^117^ ^]^ Detecting these species at their relevant concentrations and locations is of great interest for basic sciences. Due to the unique magneto‐optical property of NVs in NDs, NDs have been used to detect many different paramagnetic species^[^ ^118^ ^],^ i.e., gadolinium^[^ ^111^, ^119^ ^]^ and ferritin.^[^ ^120^, ^121^ ^]^ However, the detection of radicals with NDs was not only realized until 2020.^[^ ^13^, ^14^, ^122^, ^123^ ^]^ In the work from the group of P. Cigler,^[^ ^13^ ^]^ as illustrated in **Figure** **6**a,b,c, the NDs were coated with a poly(glycerol) shell containing covalently bound stable TEMPO radicals coupled with NV⁻ centers. The TEMPO radicals in the nanosensor are gradually reduced by ascorbates until they are completely removed. As the reaction time increases, the TEMPO radicals are removed and the *T*_1_ relaxation time is almost restored after 20 min. The oxidation process of ascorbic acid is successfully read out at the nanoscale.

In 2021, the group of R. Schirhagl succeeded in detecting free radicals in single mitochondria^[^ ^15^ ^]^ (Figure 6d–f). Two types of NDs were tested: uncoated NDs (bare‐FND), which are expected to localize in the cytosol, and anti‐VDAC2 antibody‐coated NDs (aVDAC2‐FND), which target mitochondria in macrophages. Statistically, they measured *T*_1_ times of 26 NDs in the cytosol and 36 NDs in mitochondria. The representative *T*_1_ relaxation curves for NDs in the cytosol (dark blue) and NDs in mitochondria (light blue) are shown in Figure 6e. Interestingly, the *T*_1_ times of the NDs localized in mitochondria were significantly shorter compared to cytosolic NDs. To further prove their claims, Carbonyl cyanide 3‐chlorophenylhydrazone (CCCP), an inhibitor of oxidative phosphorylation, was used to downregulate the activity of mitochondria. Low concentrations (1 × 10^−6^ and 5 × 10^−6^ m) of CCCP only slightly reduced radical generation in mitochondria. However, at 10 × 10^−6^ m CCCP, the radical generation in mitochondria was strongly enhanced. The authors also discussed other environmental parameters that could potentially disturb *T*_1_ measurements including temperature, viscosity and pH. As control, the temperature was increased from 37 to 55 °C, which only caused a negligible decrease in *T*_1_. Also viscosity of the medium did not change *T*_1_ time. When varying the pH from 3 to 8, changes in *T*_1_ time were observed, however, these changes were not considered relevant for measurements inside mitochondria, where only slight pH changes of less than one unit should occur.^[^ ^124^ ^]^ This proof‐of‐concept makes it possible to detect highly reactive free radicals in living cells with subcellular resolution. It provides us with a sophisticated nanotool to monitor the radical‐involved biological processes including immune responses. Nevertheless, in this study, the reduction in *T*_1_ times due to CCCP‐induces changes in membrane potential of the mitochondria has not addressed. Therefore, further optimization of the quantum sensor could be required to distinguish between radical formation and changes in membrane potential in mitochondria based on the experimental data.

### 4.4 Nuclear Spin Sensing with NDs and Hyperpolarization of NDs for Magnetic Resonance Imaging

**Figure 7:**
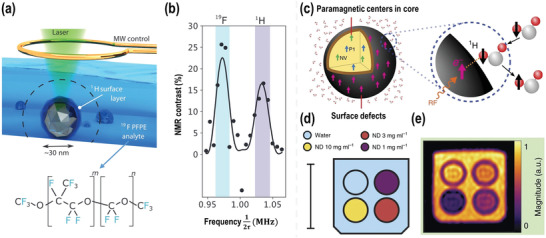
a) Illustration of an ND NMR device. 30 nm NDs (grey) with an NV center and covered by a hydrogen‐dense surface layer (dark blue) is immersed in the liquid ^19^F‐PFPE analyte solvent (light blue). The NMR signal is mainly generated by analyte nuclei close to the ND (dashed black line), enabling high spatial resolution NMR. b) Example NMR spectrum measured with a dynamical decoupling sequence. Center positions of the highlight bars correspond to the Larmor precession frequencies of ^19^F (blue bar) and ^1^H (purple bar). The widths of the bars indicate the instrumental line width of the protocol. Solid lines show fits the expected line shape. Reproduced with permission.^[^ ^138^ ^]^ Copyright 2020, American Physical Society. c) Schematic of the Overhauser effect at the ND–water interface. d) Phantom schematic. A vial of DI water (blue) and vials of HPHT 18 nm ND at concentrations of 10 mg ml^−1^ (yellow), 3 mg ml^−1^ (red), and 1 mg ml^−1^ (purple) were mounted in the phantom as shown. The surrounding volume was then filled with water (blue). Scale bar is 20 mm in length. e) OMRI images of the phantom. The color scale is normalized so that water has a magnitude of 1. Reproduced with permission.^[^ ^143^ ^]^ Copyright 2017, Nature Publishing Group.

Nuclear magnetic resonance (NMR)‐based spectroscopy and imaging are widely used in chemistry, physics, and medicine. However, conventional inductive NMR spectroscopy has limited sensitivity and only offers resolution down to the sub‐millimeter scale^[^ ^125^ ^]^ due to the low thermal nuclear spin polarization.^[^ ^126^ ^]^ Reaching nanoscale resolution appears challenging.^[^ ^127^ ^]^ As discussed above, the electronic spin of the NV center could serve as a sensitive nanoscale magnetometer under ambient conditions and in the presence of low magnetic fields. The use of NV center in bulk diamond to detect single proteins^[^ ^128^, ^129^ ^]^ enables spectral resolution at the level of chemical shifts^[^ ^130^, ^131^, ^132^ ^]^ and therefore, structures of single molecules can be detected by this approach.^[^ ^133^, ^134^, ^135^ ^]^ However, to enable single nuclear spin sensitivity, the single nuclear spin has to be confined to a small volume directly above the diamond surface. The detectability of single nuclei is very challenging as it decreases rapidly with increasing distance of the spin from the NV center below the ND surface (it falls with d^−6^ scale).^[^ ^136^, ^137^ ^]^ Using NDs containing NV centers in principle allows to position the sensor and the targeting analytes to accomplish the nanoscale measurements locally. In this way, the ND sensor could enter a biological system such as the living cell or intracellular organelles to acquire the measurements there. Recently, nanoscale NMR spectroscopy using ND was presented by Atatüre and co‐workers^[^ ^138^ ^]^ They introduced ND NMR devices and used them to detect and distinguish ^19^F and ^1^H nuclear species successfully in a sample volume of about 20^3^ nm^3^ (**Figure** **7**a,b).

In addition, ND offers the potential of label‐free nanoscale magnetic resonance imaging (MRI). However, due to the low abundance and the small gyromagnetic ratio of spin‐1/2 ^13^C nuclei in the lattice of NDs, it is ineffective to directly detect NDs in vivo. Several techniques have been developed such as conjugating paramagnetic Gd(III) to the surface of NDs^[^ ^139^ ^]^ and hyperpolarization.^[^ ^140^, ^141^, ^142^, ^143^ ^]^ One of the hyperpolarization techniques is shown in Figure 7c. Here, the spin polarization of the electron paramagnetic resonance (EPR) transition was continuously transferred from the paramagnetic centers at the ND surface to the ^1^H nuclei in the surrounding water via the Overhauser effect in the very low magnetic field (6.5 mT) by radiofrequency pulses.^[^ ^143^, ^144^ ^]^ The ^1^H MRI signal is significantly enhanced with concentration‐dependent contrast (Figure 7d,e). However, high concentrations of NDs would be required to record a detectable signal for in vivo applications, even if polarization could be performed in situ. Moreover, in case the NDs should be hyperpolarized before injection, the polarization transport losses of the NDs need to be considered. Continuous in situ hyperpolarization via the Overhauser mechanism considerably improves the theranostic capabilities of NDs and raises the possibility that MRI can be used to monitor and track ND compounds in vivo for an indefinite period.

### 4.5 Nanoscale Temperature Sensing with NDs

**Figure 8:**
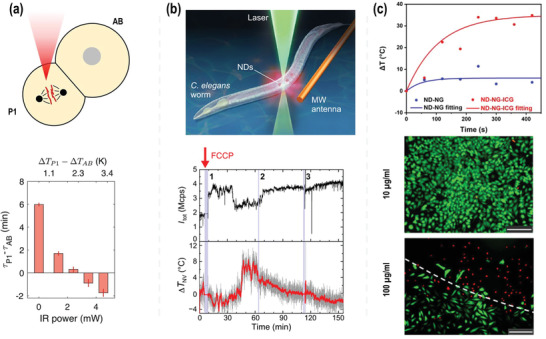
a) Observation of inversion in cell‐division order for a two‐cell embryo. P1 is selectively heated with the IR laser (upper row). Cell‐cycle time difference between the AB and P1 cells as a function of IR power (bottom row). Reproduced with permission.^[^ ^150^ ^]^ Copyright 2020, National Academy of Sciences. b) ND quantum thermometers probing inside worms. NDs are incorporated in the worms. ODMR of NV centers can be observed by applying a green laser and microwave excitation (upper row). Temperature rise inside *C. elegans* worms by chemical stimulation (bottom row). Reproduced with permission.^[^ ^151^ ^]^ Copyright 2020, American Association for the Advancement of Science. c) Change in intracellular temperature measured for NG‐NG‐ICG and ND‐NG over 420 s under NIR irradiation (upper row). Live/dead staining of HeLa cells incubated at different concentrations of ND‐NG‐ICG after 20 min irradiation using near‐infrared light (middle and bottom row). Reproduced with permission.^[^ ^99^ ^]^ Copyright 2021, American Chemical Society.

Temperature plays an important role in the various biological processes of living organisms; almost all biological activities in cells are affected by temperature, such as cell differentiation, proliferation, and death.^[^ ^145^ ^]^ Nanoscale temperature/thermal sensing is a valuable technique for elucidating the impact of temperature on biological processes at the nanoscale. Based on the temperature dependence of the zero‐field splitting, NDs could serve as robust nanoscale thermometers.^[^ ^22^, ^146^ ^]^ Increasing temperature results in a shift of the magnetic resonance to lower frequencies and an increase of their linewidth in the ODMR spectrum. Furthermore, NDs are biocompatible and physicochemically inert, and their temperature sensing property is hardly influenced by pH, ion concentration, viscosity, molecular interaction, or organic solvent.^[^ ^147^ ^]^ The first proof‐of‐principle experiment using NDs to measure temperature in human embryonic fibroblast cells was carried out by Lukin and co‐workers^[^ ^148^ ^]^ Later, the NDs were used as a thermometer for intraneuronal temperature mapping in primary cortical neurons by Simpson et al..^[^ ^149^ ^]^ Recently, many studies have been published on ND thermometers for biological applications.^[^ ^99^, ^150^, ^151^, ^152^ ^]^ **Figure** **8**a depicts a schematic of ND thermometry used to precisely probe the temperature gradient across *Caenorhabditis elegans* embryos.^[^ ^150^ ^]^ In parallel, a local infrared laser heating can manipulate the temperature on demand. In the early embryonic development of *C. elegans*, embryogenesis originates from a single P0 cell, which subsequently divides into two smaller daughter cells, AB cell and P1 cell. The AB cell is larger and develops into the anterior part of the embryo. It divides faster than the smaller, posterior P1 cell. Interestingly, the authors found that the division of P1 cells can be substantially accelerated using local heating. With the temperature increase, the division of the P1 cell accelerates and even it could even be faster than the division of the AB cell. These results indicated that both the P1 cell and AB cell followed an independent “clock” that sets the cell‐cycle timings. Moreover, they found that the cell‐division times of the P1 and the AB cells were inversed. The majority of heated embryos successfully hatched and grew into adult worms (8 of 12) with a slightly lower rate compared to the untreated worms (10 of 11). In another study reported by Fujiwara et al.^[^ ^151^ ^]^ the ND thermometer was used to monitor the thermogenic responses in adult *C. elegans* (Figure 8b) under pharmacological treatment. In the experiment, carbonyl cyanide p‐trifluoromethoxyphenylhydrazone (FCCP), another uncoupler of mitochondrial oxidative phosphorylation, was used to induce nonshivering thermogenesis, meaning heat production due to metabolic energy transformation by processes that do not involve the contraction of skeletal muscles. In the temperature response curve, at 7 min, the FCCP was added to the culture medium. At 32 min, the internal temperature within the worms started to increase. After 48 min, an additional temperature increase appeared and the total temperature increase reached 4–7 °C and it remained for about 80 min. they technically improve the microscope system by integrating a quick‐docking sample chamber, particle tracking, and an error correction filter for temperature monitoring of the moving NDs in adult *C. elegans* with a precision of ±0.22 °C. It enables us to combine the submicrometer localization with the pharmacological thermogenesis. This method will allow us to quantitatively examine biological activities based on temperature in the future.

Our group recently reported on nanodiamond theranostic for light‐controlled intracellular heating and temperature sensing within cells.^[^ ^99^ ^]^ The clinically applied photothermal agent indocyanine green (ICG) was complexed to NDs coated with a polymeric nanogel shell yielding nanodiamond‐nanogel‐indocyanine green (ND‐NG‐ICG). Under near‐infrared irradiation, the ICG produced heat resulting in a local temperature increase in HeLa cells of about 30 °C, which was measured by the ND temperature sensor (Figure 8c). Different concentrations of ND‐NG‐ICG localized within endosomal vesicles of HeLa cells were irradiated with near‐infrared light for 20 min. The results of live/dead cell staining showed that HeLa cells could tolerate intracellular temperature inhomogeneities of about 30 °C and heat needs to be accumulated to induce sufficient phototoxicity in cancer cells. In order to measure the sophisticated thermodynamic processes at distinct locations within living cells in the future, it will be necessary to introduce cell and organelle targeting groups to deliver the NDs to the desired sites such as the nucleus or mitochondria. In addition, some biological reactions may occur within the sub‐second range, which requires the development of faster measurement schemes to obtain higher signal‐to‐noise ratio (SNR) in sub‐second timescale, i.e., multipoint ODMR methods. Moreover, with the current quality of NDs, each ND shows a different split ODMR spectrum due to the different lattice strains. Therefore, measurement errors are high and calibration curves need to be recorded for each tested ND. These considerations underline the necessity to produce homogeneous NDs, where the same calibration curve fits all NDs.

### 4.6 Nanoscale Rotation Sensing with NDs

Cells are complex thermodynamic systems. Plenty of motion, diffusion and directed movements constantly occur within living cells. For example, the cargos inside cells are transported away from the nucleus along microtubules by kinesin and toward the nucleus by dynein as an illustrative example.^[^ ^153^ ^]^ Therefore, tracking dynamic intracellular processes is of emerging importance for understanding cellular dynamics and functions. To date, most tracking experiments focus on recording translational motion for example the aforementioned motion of cargos. However, to track rotational dynamics at the nanoscale in a living cell is extremely challenging, but it would be very important to elucidate many rotational dynamics, for example, traveling of molecular motors,^[^ ^154^ ^]^ ligand‐receptor interaction.^[^ ^155^, ^156^ ^]^

**Figure 9:**
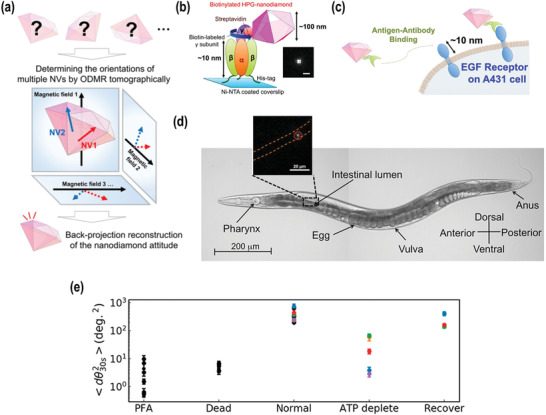
a) Determination of the orientation of a ND by ODMR. The directions of multiple NV axes are determined as projections to four different magnetic fields to reconstruct the ND orientation in the rigid body space. b) Conjugation of a ND to F1‐ATPase. Inset, fluorescence image of the NDs used in this experiment (scale bar, 1 µm). c) Conjugation of a ND to the cell membrane to study its fluctuations. d) Rotation tracking of a ND in the intestine of *C. elegans*. Orange line, the outline of the intestine; circle, nanodiamond. Reproduced with permission.^[^ ^159^ ^]^ Copyright 2020, American Chemical Society. e) Mean of squared angular displacements for the time interval of 30 s from many NDs on cells at different conditions. Reproduced with permission.^[^ ^161^ ^]^ Copyright 2021, American Chemical Society.

The spin resonances of NV centers in NDs are sensitive to tiny magnetic fields, which allows their application in magnetometry for many direct or indirect measurements, which has been discussed in Section 4.3. The spin resonance frequencies are shifted primarily by the magnetic field component projected onto the nitrogen vacancy axis. Vector magnetometry was developed to determine the relative orientation between the magnetic field and the crystallographic direction of the diamond lattice^[^ ^157^ ^]^ to measure the deformation reconstruction.^[^ ^158^ ^]^ In 2020, tracking the 3D rotational dynamics in nanoscopic biological systems was achieved by Igarashi et al.^[^ ^159^ ^]^ based on the vector magnetometry technique.^[^ ^160^ ^]^ As shown in **Figure** **9**a, the ND can contain multiple NV centers, each of which can align along with one of four different C—C bond orientations. Therefore, the orientation of the ND can be determined if the directions of at least two different NV axes in 3D space are known. The NV center is sensitive to the angle of the NV axis relative to the axis of the externally applied magnetic field.^[^ ^157^ ^]^ Therefore, the energy‐level structure of electron spins at an NV center is altered according to the pitch (*θ*), yaw (*φ*), and roll (*σ*) angles (hereafter Tait–Bryan angles) at a fixed external magnetic field by Zeeman splitting, which can be measured by ODMR technique sensitively. The authors first tracked the 3D‐rotation of a single motor protein(Figure 9b), F1‐ATPase, in the process of ATP hydrolysis after attaching the ND to the *γ*‐subunit of F1‐ATPase.^[^ ^149^ ^]^ Thereafter, a ND was conjugated to the membrane protein in living cells (Figure 9c), and the membrane fluctuation was recorded successfully. Notably, they correlated the rotational motion of the membrane protein with actin filaments in the cytoskeleton by changing the assembly/disassembly of the cytoskeletal actin filaments. They found the decreased cellular membrane dynamics originated from the increased membrane rigidity caused by the higher density of actin filaments. Moreover, they tracked the rotational dynamics of ND in the intestine of *C. elegans*. (Figure 9d)

Another notable work of rotation tracking was reported by the group of Q. Li.^[^ ^157^ ^]^ They tried to correlate the ND rotation in cell membranes with cell metabolic activities. The study was carried out by rotation tracking on paraformaldehyde (PFA) fixed cells, laser‐induced necrotized cells, and ATP‐depleted/recovered cells (Figure 9e). On the fixed cell, the rotation of the NDs dropped dramatically and almost stopped, due to the cross‐linking and immobilization of the proteins in the cells. On the laser‐induced necrotized cells, the rotation of the NDs gradually slowed down and finally stopped, because the laser‐induced necrosis that gradually shut down cellular activities. The results of the stop of rotation of the NDs on the experiments of PFA fixation and laser‐induced necrosis revealed that the rotation was not caused by the Brownian motion of the particles. In the ATP‐dependent experiments, they found that the rotation of NDs suppressed and recovered with ATP depletion and recovery. This suggests that the ATP level can be correlated to the rotation of NDs on cell membranes, which indicates that the rotation dynamics of NDs could be used as an indicator of membranes activities.

## 5 Conclusion and Outlook

Ultrasensitive detection and tracking of different environmental parameters or various species with unprecedented precision and sensitivity in the biological system will advance our understanding of living systems in general. ND quantum sensors with their high resolution down to atom sensitivity provide key features for nanoscale sensing in biology. So far, the NV centers in NDs have demonstrated their great potential for the ultrasensitive virus detection as well as for sensing pH and temperature, nuclear or electron spins in paramagnetic molecules and rotational movements. In the future, we believe that NDs could emerge as powerful tool to study biological systems under ambient conditions to provide new fundamental insights in life science. However, ND sensors are still in their infancy and many breakthroughs that have been demonstrated by physicists that still need to be realized in biological systems. The NV‐based *T*_1_ relaxometry is one of the most powerful tool to detect the radicals in biological system however, the other parameters such as pH, membrane potential may affect the *T*_1_ time as well. Therefore, more knowledge needs to be gained how different environmental features influence ND quantum sensors to clearly determine the impact of one parameter on biological processes. In the future, the following fundamental characterization of the ND quantum sensor needs to be realized to make full use of its great potential: 1) How small could be the size of the ND hosting NV center? What shortens the sensing distance and improves sensitivity? The synthesis of very small NDs with defect centers would provide less perturbations when the NDs are used to label proteins or organelles. 2) Could one create more sensitive color centers with longer sensing distance in ND suitable for quantum sensing? In order to achieve the highest sensitivity, the analytes should only be a few nanometers away from the NV center currently. 3) Would it be possible to sense pH and pH changes in living cells or even inside intracellular organelles to elucidate the pH distribution? Could one correlate local pH with biochemical processes? 4) Can we read out the content of radicals in different organelles and distinguish the certain radicals? 5) Can we detect the temperature distribution in different intracellular organelles? Can we detect the energy released by the chemical reaction in cells? 6) Can we read out the action potential in the neuron? Apart from answering these questions, NDs could also be combined with other sensors to acquire several important parameters in parallel. In particular, the combination of NDs with non‐paramagnetic species sensor (i.e., calcium sensor) could be of great interest to deepen our understanding of the roles of radicals and non‐paramagnetic species on one biological process.

## Conflict of Interest

The authors declare no conflict of interest.
